# Comparison of the Long-Term Effect of Positioning the Cathode in tDCS in Tinnitus Patients

**DOI:** 10.3389/fnagi.2017.00217

**Published:** 2017-07-28

**Authors:** Sarah Rabau, Giriraj S. Shekhawat, Mohamed Aboseria, Daniel Griepp, Vincent Van Rompaey, Marom Bikson, Paul Van de Heyning

**Affiliations:** ^1^University Department of Otorhinolaryngology and Head and Neck Surgery, Antwerp University Hospital Edegem, Belgium; ^2^Faculty of Medicine, Campus Drie Eiken, University of Antwerp Antwerp, Belgium; ^3^Section of Audiology/Health Systems, University of Auckland Auckland, New Zealand; ^4^Centre for Brain Research, University of Auckland Auckland, New Zealand; ^5^Tinnitus Research Initiative Regensburg, Germany; ^6^Department of Biomedical Engineering, City College of New York, The City University of New York New York, NY, United States

**Keywords:** neuromodulation, tDCS, electrode placement

## Abstract

**Objective**: Transcranial direct current stimulation (tDCS) is one of the methods described in the literature to decrease the perceived loudness and distress caused by tinnitus. However, the main effect is not clear and the number of responders to the treatment is variable. The objective of the present study was to investigate the effect of the placement of the cathode on the outcome measurements.

**Methods**: Patients considered for the trial were chronic non-pulsatile tinnitus patients with complaints for more than 3 months and a Tinnitus Functional Index (TFI) score that exceeded 25. The anode was placed on the right dorsolateral prefrontal cortex (DLPFC). In the first group—“bifrontal”—the cathode was placed on the left DLPFC, while in the second group—“shoulder”—the cathode was placed on the shoulder. Each patient received two sessions of tDCS weekly and eight sessions in total. Evaluations took place on the first visit for an ENT consultation, at the start of therapy, after eight sessions of tDCS and at the follow-up visit, which took place 84 days after the start of the therapy. Subjective outcome measures such as TFI, Visual Analog Scales (VAS) for loudness and percentage of consciousness of tinnitus were administered in every patient.

**Results**: There was no difference in the results for tinnitus loudness and the distress experienced between the placement of the cathode on the left DLPFC or on the shoulder. In addition, no statistically significant overall effect was found between the four test points. However, up to 39.1% of the patients experienced a decrease in loudness, measured by the VAS for loudness. Moreover, 72% of those in the bifrontal group, but only 46.2% of those in the shoulder group reported some improvement in distress.

**Conclusion**: While some improvement was noted, this was not statistically significant. Both electrode placements stimulated the right side of the hippocampus, which could be responsible for the effect found in both groups. Further research should rule out the placebo effect and investigate alternative electrode positions.

## Introduction

Tinnitus is the perception of sound in the absence of a corresponding external sound source (Eggermont and Roberts, 2004) and is a very common problem. Approximately 25.3% of the US population reports having tinnitus, while 7.9% experience tinnitus frequently (Shargorodsky et al., 2010). Tinnitus affects daily activities and can lead to a high level of distress. The literature describes several mechanisms and structures that could be involved in tinnitus. However, the findings reported cannot always be repeated and the proposed brain structures do not always match. However, it is certain that it is not only the auditory system which is responsible for inducing tinnitus (Jastreboff, 1990). Multiple non-auditory systems involved in cognition, emotion and memory play an important role (De Ridder et al., 2014).

Currently, no treatment is available that eliminates tinnitus completely. Most researchers and clinicians focus on diminishing the level of disturbance experienced and/or the loudness of the tinnitus. One method that can be used is neuromodulation, namely transcranial Direct Current Stimulation (tDCS). In the case of tDCS, the current is applied to the brain by means of two electrodes. The goal of tDCS is to influence those regions involved in tinnitus and consequently lessen the distress caused by, or the loudness of, tinnitus. Previous research has shown that bifrontal tDCS strengthens deficient inhibitory top-down mechanisms in tinnitus and interferes with the emotional processing of tinnitus (Tanaka et al., 2008; Vanneste et al., 2010). However, in the literature tDCS is reported as having a variable effect, with the percentage of responders ranging from 0% to almost 47% (Fregni et al., 2006; Vanneste et al., 2011; Song et al., 2012). The difference in outcomes might be due to different factors such as orientation of the current field, electrode positions, electrode size, stimulation duration and current intensity (Nitsche et al., 2008). Depending on the orientation of the current field, tDCS can either increase or decrease the excitability. Under the cathode, excitability is decreased due to neural hyperpolarization. However, under the anode, excitability is enhanced due to neural depolarization (Nitsche and Paulus, 2000).

The literature also reports the use of different electrode positions to treat tinnitus, with bifrontal tDCS and left temporoparietal area (LTA) tDCS most frequently applied in tinnitus patients. In bifrontal tDCS, the anode and cathode are placed on the right and left dorsolateral prefrontal cortex (DLPFC), respectively. In LTA tDCS, the anode is placed on the left temporal area. Song et al. (2012) found that LTA and bifrontal positioning produced similar results with respect to the percentage of responders and a reduction in tinnitus intensity (Song et al., 2012). Furthermore, the current intensity and duration can also play an important role. Shekhawat et al. (2013) concluded that 2 mA anodal tDCS at LTA for 20 min was the most effective setting for transient tinnitus suppression (Shekhawat et al., 2013).

Concluding, more research is needed towards parameters of tDCS to optimize the effect of it. In that interest, a computational model was calculated to predict differences in the current pathway of two placement montages, namely the “bifrontal” montage and the “shoulder” montage. The “bifrontal” group had the anode and cathode positioned over the F4 (right DLPFC) and F3 (left DLPFC) positions respectively; in the “shoulder” group, the anode electrode was placed over F4 and the cathode on the left shoulder. Considering these differences, interest was raised towards the difference in the perception outcome of the tinnitus patients undergoing tDCS using these two different montages. The objective of the present study was to compare the outcomes of the placement of the cathode on the left DLPFC vs. the shoulder.

## Materials and Methods

### Subjects

**Figure 1:**
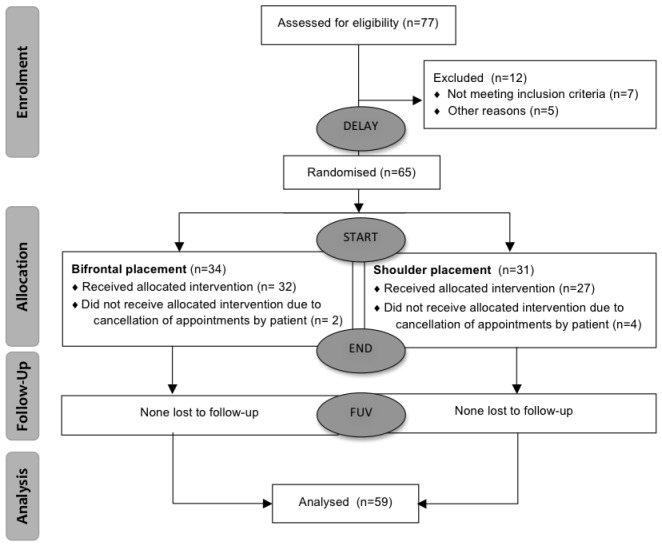
Flow chart.

Patients considered for the trial were chronic non-pulsatile tinnitus patients who had complaints for more than 3 months and a Tinnitus Functional Index (TFI) score that exceeded 25. Written informed consent was obtained from every patient (*n* = 65). The study protocol was approved by the ethical committee of Antwerp University Hospital. Of the 65 subjects, six were lost to follow-up for various reasons. The median age of the subjects was 52 years, within a range of 23 years to 70 years. The median duration of tinnitus was 28 months and fluctuated between 3 months and 264 months. In total 59 subjects—41 men and 18 women were included and randomized based on the parameters of age, TFI score, etiology, gender and degree of hearing loss. In order to create two equal groups, the MS-DOS program MINIM (by S. Evans, P. Royston and S. Day) was used to allocate the subjects by minimization. A flow diagram is presented in Figure 1.

### Transcranial Direct Current Stimulation

**Figure 2:**
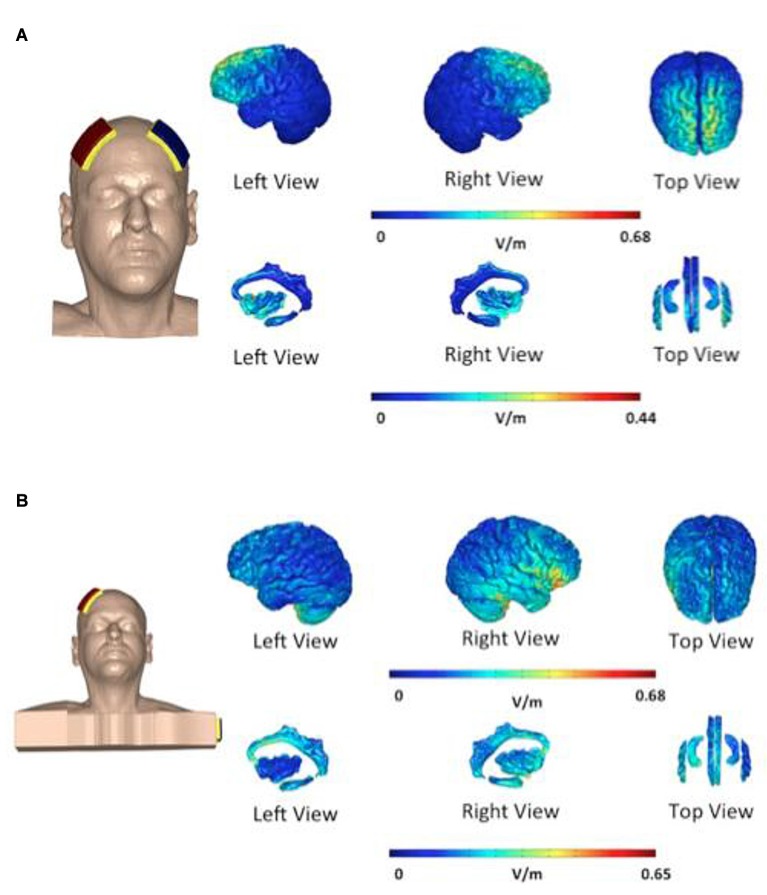
Distribution of electric field during transcranial direct current stimulation (tDCS). The electric field is shown as electric field/current density magnitude (V/m). The cathode placed on the left dorsolateral prefrontal cortex (DLPFC) **(A)** vs. the cathode placed on the shoulder **(B)**. In the bifrontal group **(A)** the DLPFC and hippocampus are stimulated. In the shoulder placement group **(B)**, the temporal lobe is stimulated in addition to the cingulate cortex and the right side of the hippocampus.

In the first group—“bifrontal”—the anode and the cathode were placed on the right and left DLPFC, respectively (F4 and F3), while in the second group—“shoulder”—the cathode was placed on the left upper arm, according to the 10–20 international system for EEG electrode placement. Each patient received two sessions of tDCS weekly and a total of eight sessions. The direct current was transferred by means of two saline-soaked pairs of surface sponge electrodes (35 cm^3^) and delivered by a specially developed, battery-driven constant current Neuroconn stimulator (neuroConn, Ilmenau, Germany), with a maximum output of 10 mA. A constant current of 2 mA was applied for 20 min with a fade-in and fade-out of 10 s. Figure 2 shows the current pattern in both cathode placements. In the bifrontal group, the DLPFC and the hippocampus are stimulated, but not the cingulate cortex. In contrast, in the shoulder placement, the temporal lobe is stimulated, in addition to the cingulate cortex and the right side of the hippocampus.

### Outcome Measures

Evaluations took place on the first visit for an ENT consultation (Delay), at the start of therapy (Start), after eight sessions of tDCS (End) and 84 days after the start of the therapy (FUV). The delay was added to test spontaneous recovery after visiting an ENT-specialist. The FUV was added to measure the long-term effect of tDCS. The flow diagram is presented in Figure 1.

The subjective outcome measures of TFI (Meikle et al., 2012; Rabau et al., 2014), Visual Analog Scales (VAS) for loudness and the Hyperacusis Questionnaire (HQ; Khalfa et al., 2002) were completed for every patient. The Dutch version of the TFI, a self-report questionnaire consisting of 25 questions, was chosen as the primary outcome measurement. A reduction of 13 points on the TFI is considered a meaningful reduction in annoyance experienced by the patient. In addition to a total score, scores of the subscales of intrusiveness, reduced sense of control, cognitive interference, sleep disturbance, auditory difficulties attributed to tinnitus, interference with relaxation, reduced quality of life and emotional distress can also be calculated (Meikle et al., 2012).

The secondary outcome measures were the VAS for loudness and the HQ. On the VAS the patient had to rate the maximum and mean loudness of their tinnitus on a scale of 0–10, with 0 indicating a very soft sound that is not audible and 10 meaning as loud as possible—the tinnitus could not be any louder. The HQ surveys the over-sensitivity to sounds, and consists of 14 items with four answer possibilities: no (0 points); yes, a little (1 point); yes, quite a lot (2 points) and yes, a lot (3 points). The maximum score is 42, with Khalfa et al. (2002) suggesting a cut-off score of 28. Patients scoring more than 28 are considered to have hyperacusis (Khalfa et al., 2002).

### Statistical Analysis

Statistical analyses were performed on the data to determine whether one placement was preferable to the other. The data were analyzed using SPSS statistical software version 22 MAC OS X (IBM; Armonk, NY, USA). Normal distribution of the data was checked using the Shapiro-Wilk test and Q-Q plots. For each of the outcome measurements, a repeated measures ANOVA was performed with test moment as within subject and group (bifrontal vs. shoulder) as between subject variable. The interaction between test moment and group were added to the model. To assess the impact of the demographic details, covariants (hearing loss and age) were added into the model. The significance level was set at *p* < 0.05. Descriptive statistics was used to compare the characteristics of the subjects.

### Computational Model

A finite element montages were generated in COMSOL multi physics 4.2 for analysis based on previous procedures (Truong et al., 2012a,b). Briefly, initially, a 3-D 1 mm*1 mm*1 mm T1 MRI of an adult male was segmented into different head regions using both automated segmentation algorithms and manual segmentation techniques available in the ScanIP software (Simpleware, Ltd., Exeter, UK) to correct for segmentation errors from the automated algorithms, add fat, segment a number of brain deep structures. 5 × 7 cm sponge pads were then placed on F4 and F3 for the “bifrontal” montage, and F4 and the left shoulder for the “shoulder” montage using ScanCAD (Simpleware, Ltd., Exeter, UK). The mesh was then generated and imported into COMSOL multi physics 4.2 (COMSOL, Inc., Burlington, MA, USA). In COMSOL, the segmented regions were assigned a conductivity (S/m): air, 10^−15^; skin, 0.465; fat, 0.025; skull, 0.01; CSF, 1.65; gray matter, 0.276; or white matter, 0.126. The electrodes were assigned a conductivity of 5.99 × 10^7^ S/m and the sponges were modeled using the conductivity of saline which is 1.4 S/m. Boundary conditions were set such that the cathode (F3 for “bifrontal”; left shoulder electrode for “shoulder”) was the ground and a total of 2 mA of current was applied from the F4 anode. The model was solved and cortical electric field magnitude was plotted for each montage to analyze the differences between the two montages.

## Results

**Figure 3:**
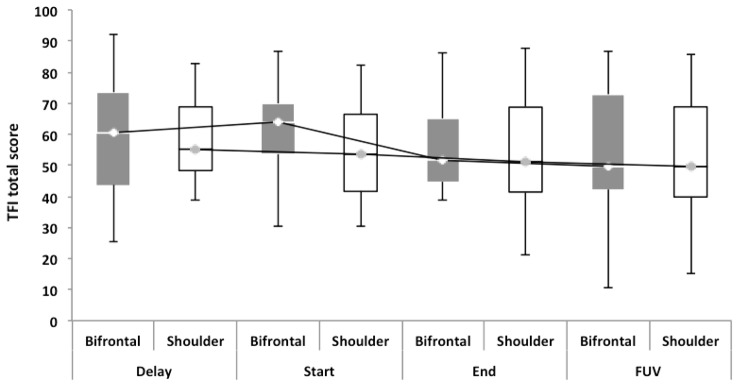
Tinnitus functional index (TFI) total score. No significant effect was found between either cathode placement and the test moments for the TFI total score.

**Figure 4:**
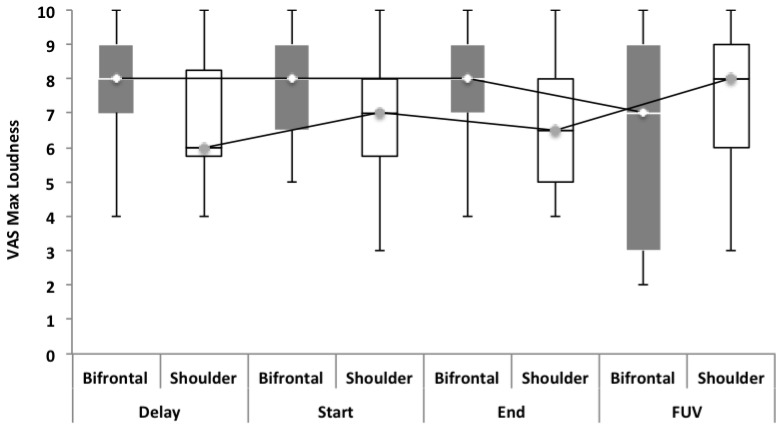
Visual analog scales (VAS) for maximum loudness. No significant effect was found between groups and the test moments for the VAS for maximum loudness.

**Figure 5:**
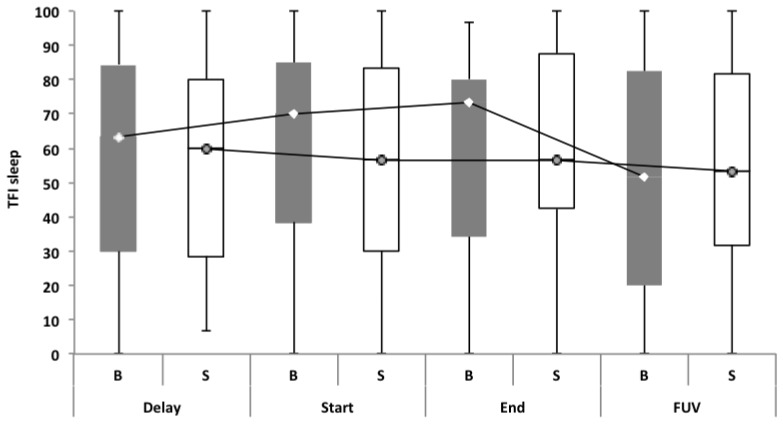
TFI subscale of sleep disturbance. In the bifrontal group, a statistically significant effect (*p* = 0.048) was found for the subscale of sleep disturbance between the Start (mean = 64.03) and the FUV (mean = 52.50) test moments.

**Figure 6:**
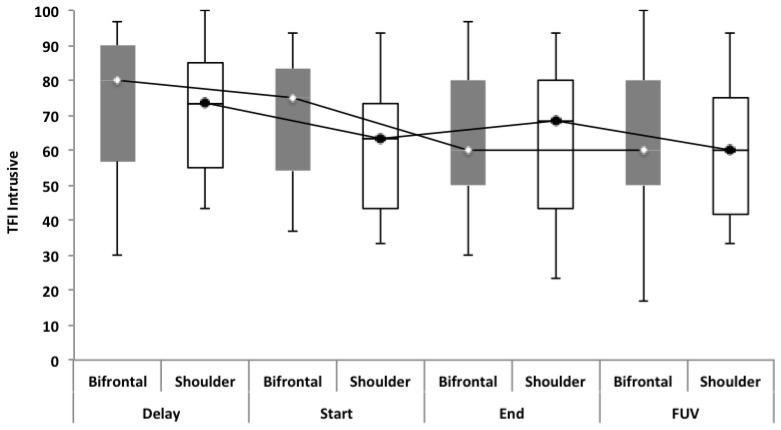
TFI subscale of intrusiveness. Concerning the shoulder group, a statistically significant effect was found for the subscale of intrusiveness (*p* = 0.040) between the Delay (mean = 72.50) and the FUV (mean = 62.50) test moments. The effect found cannot be attributed to the tDCS therapy, but to the spontaneous recovery or placebo effect found between the Delay (median = 73.30) and Start (median = 63.33) test moments.

Repeated measures showed no statistically significant difference between both groups with regard to the TFI, the subscales of the TFI, the VAS for maximum and mean loudness, the percentage of consciousness, or problems experienced falling asleep and waking up during the night (*p* > 0.05). Looking at the overall effect, no significant effect was found between the test moments for the TFI total score (Figure 3), the TFI subscales of intrusiveness, cognitive interference, sleep disturbance, auditory difficulties, quality of life and emotional distress, or with the maximum (Figure 4) and mean VAS for loudness and percentage of consciousness (*p* > 0.05). For the subscale of sleep disturbance (*p* = 0.048), a statistically significant effect was found between the Start (mean = 64.03) and the FUV (mean = 52.50) test moments in the bifrontal group (Figure 5). Concerning the shoulder group, a statistically significant effect was found for the subscale of intrusiveness (*p* = 0.040) between the Delay (mean = 72.50) and the FUV (mean = 62.50) test moments. The results are shown in Figure 6. No statistically significant effect of the demographic details age and hearing loss was found on the outcome measurements (*p* > 0.05).

Focusing on the measurements before and after the eight sessions of tDCS, we found that 26.8% (*n* = 7) of the bifrontal group and 37.5% (*n* = 9) of the shoulder group experienced an improvement of 1 or more on the VAS for maximum loudness, while 30.6% (*n* = 8) of the subjects in the bifrontal group and 39.1% (*n* = 9) of the subjects in the shoulder group showed an improvement on the VAS for mean loudness. With respect to the change in TFI scores, 16% (*n* = 4) of the bifrontal group and 8.4% (*n* = 2) of the shoulder group showed a clinically significant improvement of more than 13 points, while 56% (*n* = 14) of the bifrontal group and 37.8% (*n* = 9) of the shoulder group showed an improvement of less than 13 points on the TFI.

## Discussion

The present study failed to find any difference between the electrode placements with respect to their effects on tinnitus distress and tinnitus loudness. One possible explanation for this may be the limited focus of the tDCS, due to the large electrodes used (Nitsche et al., 2008). The lack of focus prevents the stimulation of a specific area of the brain. Moliadze et al. (2010) have suggested that an extracephalic reference electrode has the potential to better focus stimulation (Moliadze et al., 2010), but direct proof is lacking. In contrast, Parazzini et al. (2012) argued that if the anode and cathode are placed on the far sides of the brain this results in a more widely spread current/electric field (Parazzini et al., 2012). Bikson has studied the role of the position of the return electrode, finding that the repositioning of the return electrode from the contralateral forehead to the upper arm caused the relocation of the current from the frontal regions to the more posterior regions of the brain (Bikson et al., 2010). This finding is consistent with our current pattern, as presented in Figure 2. Tissue resistance and altered current flow can also play an important role (Nitsche and Paulus, 2011). Moliadze et al. (2010) claimed that higher current intensity is required to induce identical aftereffects in comparison to bicephalic electrode placement (Moliadze et al., 2010). In relation to bicephalic electrode placement, Shekhawat et al. (2013) concluded that 2 mA anodal tDCS at the LTA for 20 min was sufficient (Shekhawat et al., 2013). Further research should determine whether 2 mA is sufficient to induce aftereffects in the case of extracephalic reference electrode placement and the best dose-response ratio with this electrode position.

No statistically significant overall effect was found between the four test moments. Only for the TFI subscale of sleep disturbance in the bifrontal group was a significant effect found between the Start (median = 70.00) and FUV (median = 51.67) test moments. The statistically significant difference found for the subscale of intrusiveness in the shoulder group cannot be attributed to the tDCS therapy, but to spontaneous recovery or a placebo effect between the Delay (median = 73.30) and Start (median = 63.33) test moments. To rule out a placebo effect, we recommend including a control group in the study protocol for further research. The percentage of responders varied from 26.8% to 39.1% for tinnitus loudness. For the outcome measurement of distress, a clinically significant effect was only found in 16% and 8.4% of the subjects in the bifrontal and shoulder groups respectively. None of these results were statistically significant. The results confirm previous findings reported in the literature, which suggest no long-term effects on distress. However, a positive transient effect of bifrontal tDCS has been reported for tinnitus intensity (Garin et al., 2011; Frank et al., 2012). The present study failed to repeat these results. Most studies have reported a positive effect of tDCS on tinnitus loudness and distress immediately after a tDCS session (Vanneste et al., 2010; Vanneste and De Ridder, 2011; Joos et al., 2014); however, in this study, we focused on the long-term effect.

Another explanation for the lack of statistically significant results may be related to the factors that cause the variance of responders. For tDCS to be considered useful in a clinical setting, it would be interesting to know what causes this variance in the percentage of responders. Further research should also determine what might improve the administration of tDCS to attain clinically significant outcomes. High definition tDCS seems to be a promising alternative in this respect (Shekhawat et al., 2016).

While some improvement was found in both groups, the difference was not statistically significant. This finding would suggest that the area that was stimulated in both groups can be hold responsible for this improvement, namely the right side of the hippocampus, which is the part of the brain that is involved in learning and memory processes. Jastreboff (1990) has pointed out that in addition to the auditory system, the limbic system, including the hippocampus, is responsible for the preseverance of tinnitus. The gating model proposed by Rauschecker et al. (2010) also makes predictions about the structures involved in tinnitus. Although the hippocampus is not a part of the model presented, this structure may be involved in tinnitus but not sufficient to cause tinnitus on its own (Rauschecker et al., 2010; Adjamian et al., 2014). The positioning of an electrode such that it influences the structures involved in the proposed gating model would be an interesting topic for further research. In this study we did not investigate the underlying structures activated during the tDCS stimulation during the two montages (cathode on L DLPFC and shoulder). However, in future it would be insightful to incorporate MRI scans to investigate the neurophysiological structures involved during the tDCS. Another possible explanation is that the effect found can be attributed to a placebo effect. Further research should include a control group to rule out the placebo effect and reveal the real effect of tDCS.

## Conclusion

There was no significant difference in outcomes between the electrode placements. Both placements stimulated the right side of the hippocampus, which could be responsible for the effect found in both groups. Further research should rule out a placebo effect, while alternative electrode positions, as well as high definition tDCS, could reveal the effects of stimulating brain structures that are involved in the gating model proposed by Rauschecker et al. (2010).

## Author Contributions

SR, VVR and PV designed and set up the study protocol. SR collected and analyzed the data. SR and MB wrote the manuscript. MA and DG designed the model of the distribution of the electric field of tDCS. GSS, VVR, DG, MA and MB reviewed the manuscript.

## Conflict of Interest Statement

The authors declare that the research was conducted in the absence of any commercial or financial relationships that could be construed as a potential conflict of interest. The reviewer YSY and handling Editor declared their shared affiliation, and the handling Editor states that the process nevertheless met the standards of a fair and objective review.
